# A Convenient Colorimetric Bacteria Detection Method Utilizing Chitosan-Coated Magnetic Nanoparticles

**DOI:** 10.3390/nano10010092

**Published:** 2020-01-02

**Authors:** Thao Nguyen Le, Tai Duc Tran, Moon Il Kim

**Affiliations:** Department of BioNano Technology, Gachon University, 1342 Seongnamdae-ro, Sujeong-gu, Seongnam, Gyeonggi 13120, Korea; thaonguyen65949@gmail.com (T.N.L.); tdtai.151294@gmail.com (T.D.T.)

**Keywords:** colorimetric bacteria detection, chitosan-coated magnetic nanoparticles, peroxidase-like activity, *Escherichia coli*, *Staphylococcus aureus*

## Abstract

An effective novel strategy to detect bacteria is promising because it may improve human health by allowing early diagnosis and timely treatment of bacterial infections. Here, we report a simple, reliable, and economical colorimetric assay using the peroxidase-like activity of chitosan-coated iron oxide magnetic nanoparticles (CS-MNPs). When CS-MNPs are incubated with a sample containing bacterial cells such as the gram-negative *Escherichia coli* or the gram-positive *Staphylococcus aureus*, the negatively-charged bacterial membrane interacts with positively-charged chitosan on the surface of CS-MNPs, thus resulting in significant reduction of their peroxidase-like activity presumably by a hindrance in the accessibility of the negatively charged substrate, 2-2′-azino-bis(3-ethylbenzothiazoline-6-sulfonic acid) diammonium salt (ABTS) to the positively-charged CS-MNPs. This simple colorimetric strategy allowed the rapid detection of bacterial cells down to 10^4^ CFU mL^−1^ by the naked eye and 10^2^ CFU mL^−1^ by spectrophotometry within 10 min. Based on the results, we anticipate that the CS-MNPs-based assay has great potential for the on-site diagnosis of bacterial infections in facility-limited or point-of-care testing (POCT) environments.

## 1. Introduction

Bacterial contamination in myriad environments has become a serious threat to human life as it can cause various diseases. Among many pathogenic bacteria in water, food, or pharmaceutical products, gram-negative *Escherichia coli* and gram-positive *Staphylococcus aureus* are two major bacterial species causing disease including diarrhea, anemia, skin infections, orbital cellulitis, urinary tract infections, and eventually death [1,2,3]. According to the World Health Organization (WHO), infectious diseases by food-borne pathogenic bacteria caused 240,000 deaths globally in 2010. Considering the seriousness of bacterial contamination, a simple, rapid, reliable, sensitive, and early-screening method to detect bacteria is critically important to conduct early-diagnosis as well as timely treatment to effectively prevent and cure the disease [4].

There are many methods for bacteria detection. The plate colony counting method is considered as a “gold standard” because it provides a direct enumeration of bacteria through cultivation under standard conditions. However, it inevitably requires specific facilities for microbial cell cultivation and skilled personnel, as well as a long-time analysis of up to several days for enrichment, plating and isolation, and identification [5]. Other techniques including polymerase chain reaction (PCR) and immunological methods such as enzyme-linked immunosorbent assay (ELISA) have been widely used for bacterial detection based on their ability to overcome such time limitations with high detection sensitivity and selectivity. However, these methods are conducted with specialized instruments, complicated sample treatment and analytic procedures, and well-trained individuals with the appropriate expertise. More importantly, these methods cannot be conducted on-site, critically restricting their use in limited facilities or point-of-care testing (POCT) environments [6,7].

To develop bacteria detection methods particularly suitable for on-site detection, colorimetric techniques have attracted increasing attention due to their easy operation and visual detection without any instrumentation/expertise [8,9]. Several enzymatic assays have been reported for the colorimetric detection of bacteria, for example, T7 bacteriophages carrying the lacZ operon were used to infect *E. coli*, allowing the overexpression of lacZ-dependent β-galactosidase to catalyze a colorimetric reaction depending on the amount of *E. coli* [10]. Glucose oxidase-mediated colorimetric detection methods were also reported for broad-spectrum bacteria carrying out glucose metabolism [11]. These enzymatic methods provide simple colorimetric identification of bacteria, however, specific biological elements like phage-induced systems are often difficult to apply for broad-spectrum bacteria detection. Also, enzyme instability has significantly hindered its practical utilization.

To overcome these limitations, diverse nanomaterials such as nanoparticles, nanorods, nanowires, and carbon nanostructures have been used because of their excellent physicochemical properties [12,13]. Noble metal nanoparticles such as those composed of gold and silver have been investigated based on their unique optical properties inducing a distinct color change by aggregation or chemical reaction on their surface [14,15]. They produce a rapid and sensitive colorimetric response to target bacteria, however, they generally require laborious surface modification with biomolecules such as DNA, RNA, and antibodies, and are often quite sensitive to experimental conditions causing false positives. Several peroxidase-mimicking nanomaterials including gold nanoparticles, graphene oxide, and iron oxide magnetic nanoparticles (MNPs) were also explored for the specific detection of pathogenic bacteria, as they exhibit noticeable enzyme activity with extremely high stability and they can be mass-produced at low cost [16,17,18]. Like natural peroxidase, these nanomaterials can decompose H_2_O_2_ into an OH· radical that can oxidize peroxidase substrates such as 3,3′,5,5′-tetramethylbenzidine (TMB) or 2-2′-azino-bis (3-ethylbenzothiazoline-6-sulfonic acid) diammonium salt (ABTS) yielding blue and green color, respectively [18,19]. Typically, pathogen-specific receptors like antibodies or DNA aptamers are conjugated on the surface of the nanomaterials and they are used to detect bacteria via sandwich-type assay procedures [20,21]. Several reports demonstrated that the sandwich-type colorimetric assay is successful to detect bacterial cells, however, it requires tedious surface modification steps as well as many experimental procedures [22,23,24]. A label-free detection method using MNPs was also recently reported. In the assay, DNA aptamer molecules that have a specific affinity for *Salmonella typhimurium* were first adsorbed onto the surface of MNPs by electrostatic interaction resulting in the inhibition of the peroxidase-like activity of the MNPs [25]. Through the addition of sample solutions containing target *S. typhimurium*, aptamers bound on MNPs strongly interact with bacteria and are detached from the MNPs, thereby increasing their peroxidase activity. Using this strategy, target *S. typhimurium* is detected through observing the green color from the oxidation of the ABTS substrate; however, its sensitivity is too low and the limit of detection is 7.5 × 10^4^ colony-forming unit (CFU) mL^−1^, presumably due to the insufficient control of the aptamer affinity between MNPs and bacterial cells [25]. Therefore, it is desirable to elaborately control the peroxidase activity of MNPs depending on the number of bacterial cells, to achieve higher sensitivity for practical on-site colorimetric detection.

Herein, we used chitosan, a polycationic biopolymer containing abundant amine groups in its backbone, to modify the surface of MNPs (CS-MNPs). Positively charged chitosan has been proven to show a high affinity toward broad-spectrum bacteria, majorly due to the negative surface charge as well as outer-core lipopolysaccharides of the bacterial membrane [26]. Based on the high affinity of chitosan toward broad-spectrum bacteria, we hypothesized that bacterial cells would effectively bind to CS-MNPs, thereby inhibiting their peroxidase-like activity, which would produce a decreased colorimetric response. Gram-negative *E. coli* and gram-positive *S. aureus* were successfully determined at 10^4^ CFU mL^−1^ by the naked eye and at 10^2^ CFU mL^−1^ by spectrophotometry within 10 min. Scanning electron microscopy (SEM) and zeta potential analyses were used to understand the binding mechanism between CS-MNPs and the bacterial cells.

## 2. Experimental Section

### 2.1. Materials

Chitosan (CS), iron (III) chloride hexahydrate (FeCl_3_∙6H_2_O), iron (II) chloride tetrahydrate (FeCl_2_∙4H_2_O), sodium hydroxide, phosphate-buffered saline (PBS), ABTS, sodium acetate, hydrogen peroxide (H_2_O_2_ 30% *w*/*v*), acetic acid, ethanol, and glutaraldehyde (25% *w*/*v*) were purchased from Sigma-Aldrich (Milwaukee, WI, USA). The Luria-Bertani (LB) broth and agar powder were purchased from Becton, Dickinson and Company (Franklin Lakes, NJ, USA). All solutions were prepared with deionized water purified with a Milli-Q Purification System (Millipore, MA, USA).

### 2.2. Synthesis of MNPs and CS-MNPs

MNPs were synthesized using a reported co-precipitation method [27]. Briefly, 1 M sodium hydroxide was added to the aqueous mixture of 0.25 M FeCl_2_ and FeCl_3_ (Fe^3+^/Fe^2+^ = 2) until pH turned 10. Then, the mixture was heated at 80 °C for 40 min under stirring until color changed from bright brown to black. The black precipitates were washed several times with deionized water and ethanol to remove salt residues. The resulting MNPs were dried under vacuum at 70 °C overnight.

MNP surfaces were coated with chitosan to produce CS-MNPs as follows: MNPs (50 mg) were dispersed in distilled water (50 mL) under sonication for 30 min followed by a slow addition of 2% *w*/*v* chitosan solution in 1% *v*/*v* acetic acid while stirring at 1000 rpm at 50 °C. After 12 h reaction, the resulting CS-MNPs were collected by magnetic separation after multiple rinses using water and ethanol to remove free chitosan. The resulting CS-MNPs were dried at 60 °C under vacuum overnight [28].

### 2.3. Characterization of MNPs, CS-MNPs, and Bacteria with Nanoparticles

The size, morphology, and elemental composition of MNPs and CS-MNPs were analyzed by SEM images on a Magellan 400 microscope (FEI Co., Eindhoven, The Netherlands) and an energy dispersive X-ray spectrometer (EDS). Nanoparticle samples for SEM were prepared by placing a drop of suspension on a polished wafer and dried overnight at room temperature. Zeta potential analysis was carried out using a Zetasizer Nano-ZS (Malvern Co., Worcestershire, UK). Fourier-transform infrared (FT-IR) spectra and X-ray diffraction (XRD) patterns of MNPs, CS-MNPs, and free chitosan were obtained using an FT-IR spectrophotometer (FT/IR-4600, JASCO, Easton, MD, USA) and an X-ray diffractometer (D/MAX-2500, Rigaku Corporation, Tokyo, Japan), respectively.

The interaction of MNPs and CS-MNPs with *E. coli* or *S. aureus* was also analyzed by SEM and zeta potential analysis. For SEM, bacteria were first grown in LB media to log phase (OD_600 nm_ ~ 0.6). Then, bacterial cells were centrifuged at 8000 rpm for 3 min, washed several times with PBS, and serially diluted in PBS to obtain 10^5^ CFU mL^−1^ suspensions. The cells were treated with 200 μg mL^−1^ MNPs or CS-MNPs for 6 h. After treatment, the mixture was centrifuged at 8000 rpm for 3 min and rinsed twice with PBS. Subsequently, cells were fixed with 2.5% glutaraldehyde in PBS for 4 h and further rinsed twice in PBS. After fixation, cells were subjected to dehydration in a series of ethanol solutions with increasing concentrations (20%, 30%, 40%, 50%, 60%, 70%, 80%, 90%, and pure ethanol) for 10 min in each step. Finally, cells were added dropwise onto a silicon wafer and air-dried overnight for SEM analysis.

### 2.4. Selected Bacteria and Culture Conditions

*E. coli* WT3110 strain and *S. aureus* ATCC 25923 strain were used for the bacterial detection experiments. Bacteria were grown in LB medium at 37 °C and 200 rpm in a shaking incubator (SI-7820, Optima, Japan). Then, cultured bacteria were centrifuged at 8000 rpm for 5 min and resuspended in deionized water for the colorimetric detection experiments. The number of bacteria was determined by colony counting on LB-agar plates after incubation for 24 h at 37 °C.

### 2.5. Colorimetric Bacteria Detection Using CS-MNPs

A 1 × 10^9^ CFU mL^−1^ bacterial suspension was prepared and diluted to various concentrations. Colorimetric bacteria detection was carried out in transparent tubes or 96-well plates containing MNPs or CS-MNPs (0.1 mg mL^−1^), H_2_O_2_ (2.5 mM), ABTS (6 mM), and a bacterial cell suspension in sodium acetate buffer (50 mM, pH 4.0) at different concentrations. After 10 min, nanoparticles and bacterial cells were removed by centrifugation at 12,000 rpm for 5 min. The supernatant solution was used to obtain images and the corresponding absorption spectra or absorption intensity at 417 nm on a microplate reader (Synergy H1, BioTek, VT, USA).

## 3. Results and Discussion

### 3.1. Preparation and Structural Characterization of MNPs and CS-MNPs

**Figure 1:**
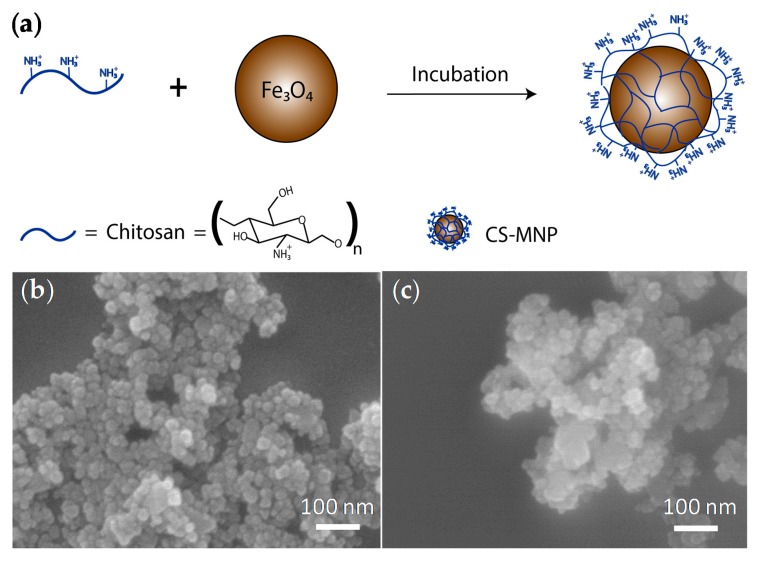
(**a**) Schematic illustration of chitosan coating the surface of iron oxide magnetic nanoparticles to prepare CS-MNPs. SEM image of (**b**) MNPs and (**c**) CS-MNPs.

Because positively-charged chitosan has been known to present a high electrostatic affinity toward negatively-charged bacterial cell membranes, MNPs were first coated with chitosan to prepare CS-MNPs (Figure 1a). Chitosan was dissolved in acidic pH conditions for the protonation of its amine groups [29]. The morphology and size of the resulting MNPs and CS-MNPs were then analyzed by SEM images (Figure 1b,c). Both MNPs and CS-MNPs presented spherical shapes with a nearly-uniform diameter of around 30 nm.

**Figure 2:**
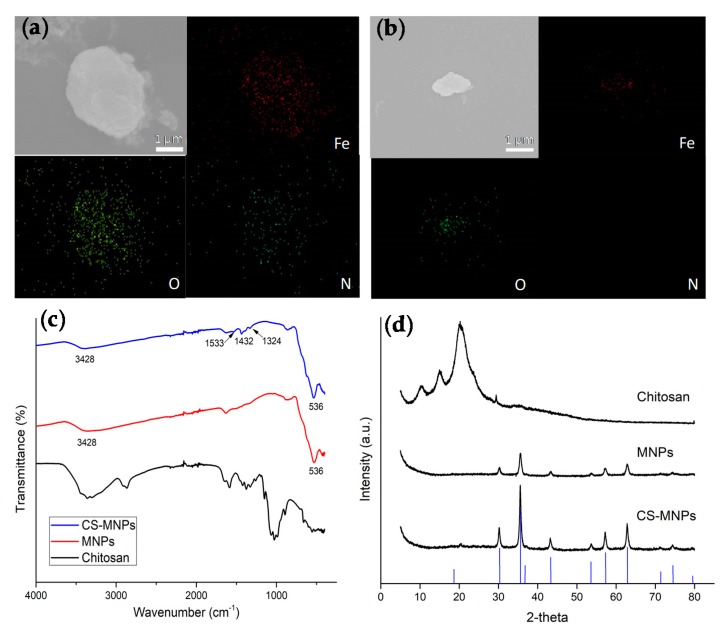
SEM images of (**a**) CS-MNPs and (**b**) MNPs with their corresponding EDS maps of Fe, O, and N. (**c**) FT-IR spectra and (**d**) XRD spectra for CS-MNPs, MNPs, and chitosan.

For the bacterial detection experiments, the surface charge of bare MNPs was +2.94 mV, and gradually increased as the concentration of chitosan also increased, as positively-charged chitosan has good adhesion to the hydroxyl group of MNPs (Figure S1) [30]. The SEM-EDS elemental mapping images of Fe, O, and N for CS-MNPs indicate the homogeneous distribution of each element throughout the aggregated nanoparticles, which are the main components of MNPs and chitosan (Figure 2a). In contrast, bare MNPs did not show any N, clearly revealing the absence of chitosan (Figure 2b). Chemical compositions were also confirmed by FT-IR analyses (Figure 2c). Typical characteristic peaks corresponding to the Fe-O bond were observed at 536 cm^−1^ for both MNPs and CS-MNPs, and furthermore, the peaks at about 3300–3340 cm^−1^ represent O-H stretching vibration, demonstrating the presence of iron oxide nanoparticles [31]. For CS-MNPs, there were peaks at 1324 and 1432 cm^−1^ indicating C-N stretching, and 1533 cm^−1^ indicating the bending vibration of NH_3_^+^, clearly showing the presence of chitosan [32,33,34]. These characteristic peaks were not detected in MNPs because of the absence of chitosan. XRD patterns also showed six characteristic peaks for Fe_3_O_4_ crystal with the indices (220), (311), (400), (422), (511) and (440) (Figure 2d) [35]. The peaks detected at around 20° for CS-MNPs are closely associated with chitosan.

### 3.2. Rapid and Sensitive Colorimetric Bacteria Detection Using CS-MNPs

**Figure 3:**
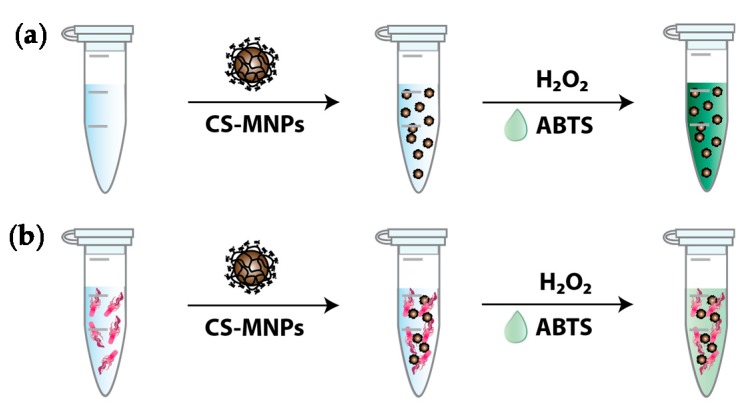
Schematic representation of the CS-MNPs-based colorimetric bacteria detection (**a**) in the absence or (**b**) in the presence of bacterial cells.

The novel colorimetric assay for the detection of broad-spectrum bacteria is illustrated in Figure 3. The procedure involved the simple incubation of CS-MNPs with a sample solution for just 10 min at room temperature to detect broad-spectrum bacteria. When there were no bacteria in the sample solution, CS-MNPs exhibited their original peroxidase-like catalytic activity and a dense green color from the oxidation of ABTS. On the contrary, in the presence of bacteria, CS-MNPs were bound to their surface through electrostatic interactions resulting in a decreased available surface area for catalytic events with increased hindrance in the accessibility of the negatively-charged ABTS to the positively-charged CS-MNPs and thereby leading to the reduced peroxidase-like activity of CS-MNPs. Overall, assay samples containing bacterial cells displayed significantly lowered colorimetric responses while those without bacteria produced an intense colorimetric response. As this color-generating reaction from the ABTS oxidation takes only 10 min at room temperature, we expect this assay to be used for rapid but effective detection of broad-spectrum bacteria on site.

**Figure 4:**
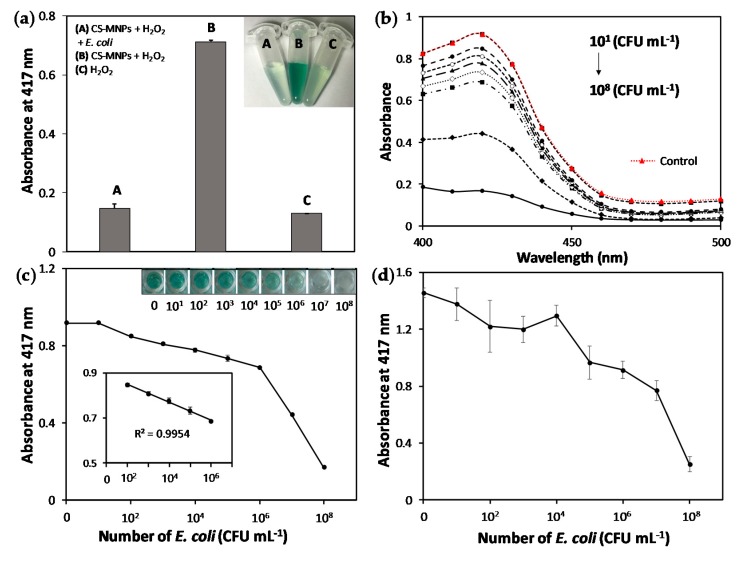
(**a**) Absorption intensity of the reaction leading to ABTS oxidation using CS-MNPs in the presence or absence of *E. coli* (10^8^ CFU mL^−1^). Real photograph of the reaction solution is shown in the inset; (**b**) Absorption spectra of the reaction tube at various *E. coli* concentrations ranging from 10^1^ to 10^8^ CFU mL^−1^. (**c**) Absorption intensity of solutions obtained from the CS-MNPs-based assay at various *E. coli* concentrations. Real photograph of the reaction solution is shown in the inset; (**d**) Absorption intensity of solutions obtained from the bare MNPs-based assay at various *E. coli* concentrations.

We first found out that CS-MNPs retained over 80% of the original peroxidase activity of bare MNPs (Figure S2), which was enough to produce a colorimetric response. As expected, the catalytic activity of CS-MNPs was significantly reduced in the presence of gram-negative *E. coli* (Figure 4a), probably because of the increased hindrance in the accessibility of the ABTS substrate to CS-MNPs. As *E. coli* concentration increased from 10^1^ to 10^8^ CFU mL^−1^, the absorbance at 417 nm corresponding to the oxidized ABTS gradually decreased (Figure 4b). From the calibration plots, *E. coli* might be spectrometrically determined at concentrations as low as 10^2^ CFU mL^−1^ with a linear range from 10^2^ to 10^6^ CFU mL^−1^ (Figure 4c). Even without any detection instrument, the presence of *E. coli* in the sample solution could be visibly discriminated by the naked eye at 10^4^ CFU mL^−1^. When we used bare MNPs rather than CS-MNPs for the same *E. coli* detection experiments, their peroxidase activity was not efficiently inhibited by the presence of bacterial cells, probably due to the inefficient binding between bacteria and MNPs, and furthermore, there were significant variations in the absorbance, which made bacterial quantification difficult (Figure 4d).

**Figure 5:**
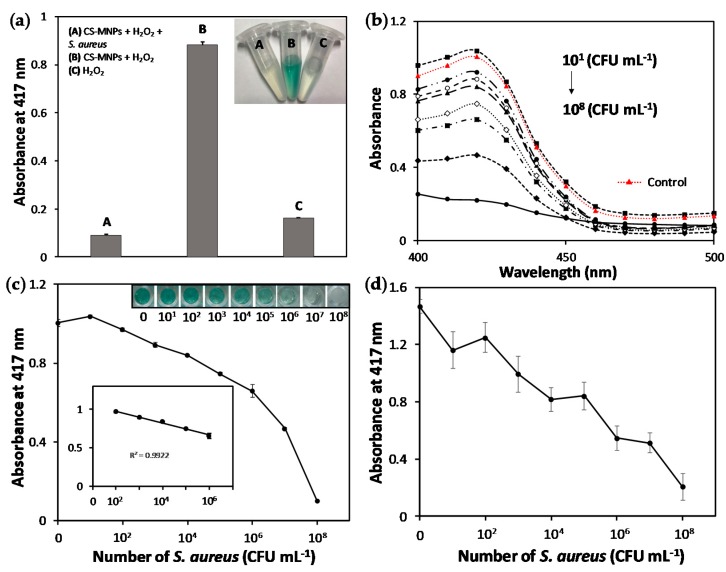
(**a**) Absorption intensity of the reaction leading to ABTS oxidation using CS-MNPs in the presence or absence of *S. aureus* (10^8^ CFU mL^−1^). Real photograph of the reaction solution is shown in the inset; (**b**) Absorption spectra of the reaction tube at various *S. aureus* concentrations ranging from 10^1^ to 10^8^ CFU mL^−1^; (**c**) Absorption intensity of solutions obtained from the CS-MNPs-based assay at various *S. aureus* concentrations. Real photograph of the reaction solution is shown in the inset; (**d**) Absorption intensity of solutions obtained from the bare MNPs-based assay at various *S. aureus* concentrations.

Although chitosan has a higher affinity toward gram-positive bacteria than to gram-negative ones due to their relatively higher positive charge density, our CS-MNPs-based colorimetric bacteria detection resulted in similar sensitivity for both types of bacteria [36,37]. Absorbance (417 nm) was also significantly decreased in the presence of gram-positive *S. aureus* due to their binding-mediated inhibition of CS-MNPs’ peroxidase activity (Figure 5a). Gradually reduced colorimetric signals were also produced from the samples containing increased *S. aureus* concentrations ranging from 10^1^ to 10^8^ CFU mL^−1^ (Figure 5b). The linear range was established from 10^2^ to 10^6^ CFU mL^−1^ and the limit of detection was determined as 10^2^ CFU mL^−1^ (Figure 5c). Similarly, *S. aureus* cells at 10^4^ CFU mL^−1^ were discriminated from the control sample without bacteria by the naked eye. With the bare MNPs, although peroxidase activity was marginally inhibited at concentrations over 10^6^ CFU mL^−1^, there were large variations in the absorbance, which was not suitable for bacterial quantification (Figure 5d).

### 3.3. Demonstration of the Interaction between CS-MNPs and Bacteria

**Figure 6:**
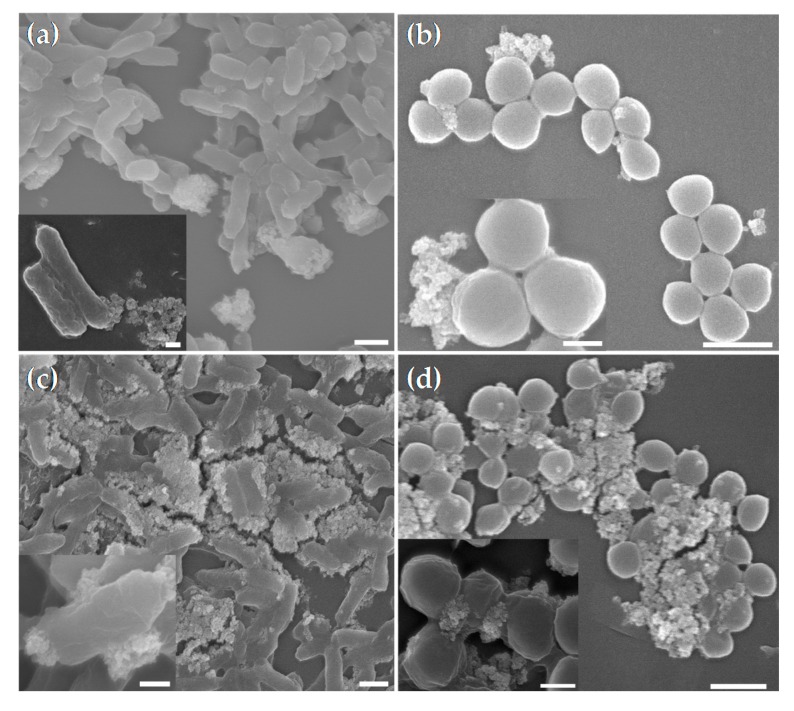
SEM images of (**a**) MNPs incubated with *E. coli*. (**b**) MNPs incubated with *S. aureus*. (**c**) CS-MNPs incubated with *E. coli* and (**d**) CS-MNPs incubated with *S. aureus*. Scale bars indicate 1 µm. In the insets, scale bars indicate 0.3 µm.

We hypothesized that the electrostatic interaction between positively-charged CS-MNPs and negatively-charged bacteria explained the reduced colorimetric signal by hindering substrate accessibility to the CS-MNPs. To test the hypothesis, SEM analyses were conducted and the results showed the increased binding of CS-MNPs to bacteria compared to bare MNPs (Figure 6). In the case of bare MNPs, a lower number was detected on the surface of both *E. coli* and *S. aureus* indicating a relatively weak interaction between MNPs and bacteria. In contrast, CS-MNPs resulted in a much more efficient binding on the surface for both types of bacteria, showing a stronger attraction, mainly due to the protonated amine group of chitosan at acidic pH conditions with the anionic membrane of bacteria.

**Figure 7:**
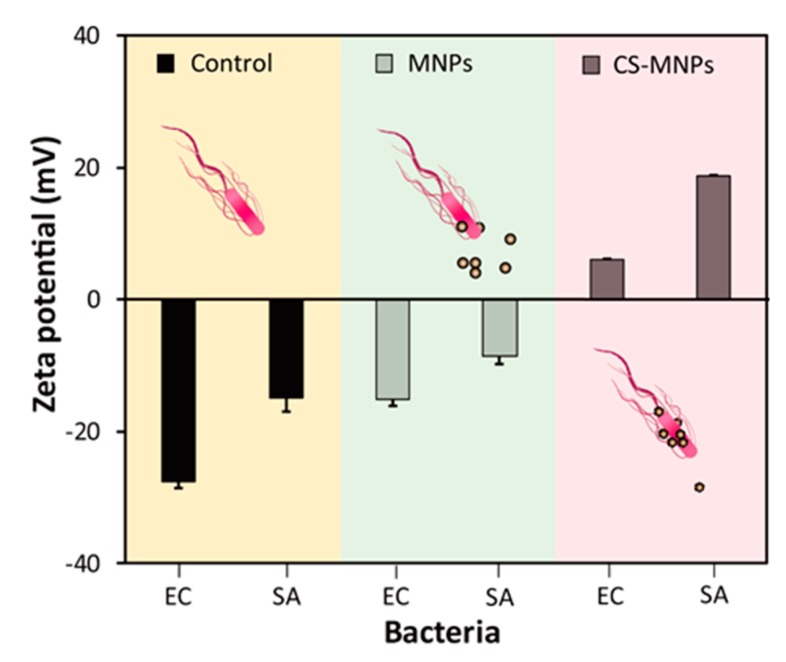
Zeta potential analyses of bacteria only, bacteria with bare MNPs, and bacteria with CS-MNPs. *E. coli* (EC) and *S. aureus* (SA) were used in the experiments.

Surface zeta potentials of bacteria with or without MNPs and CS-MNPs were also analyzed to clearly understand the role of surface charge on the interaction between bacteria and nanoparticles (Figure 7). The surfaces of both bacterial strains were found to have a negative charge, as previously observed [36]. When bacteria were incubated with MNPs, the surface charges marginally increased but still had negative values, as bare MNPs showed slightly-positive zeta potentials. Finally, by incubating with CS-MNPs, the surface potential increased notably and yielded positive values for both bacterial strains, revealing the efficient binding of CS-MNPs to the surface of bacteria. As expected, both bacteria incubated with free CS without MNPs also showed notably increased surface zeta potentials, due to the effective interaction with positively-charged CS (Figure S3). This investigation demonstrated that positively-charged CS-MNPs effectively interacted with negatively-charged bacteria, which critically contributed to the reduced catalytic activity of CS-MNPs.

## 4. Conclusions

In summary, a simple, rapid, inexpensive, and instrument-free colorimetric bacteria detection method has been successfully developed based on the efficient electrostatic interaction between CS-MNPs and bacterial cells, as well as the concomitant inhibition of the peroxidase activity of CS-MNPs. This assay can be conveniently conducted and analyzed by monitoring the green color reduction within 10 min. Both gram-negative *E. coli* and gram-positive *S. aureus* were successfully quantified at concentrations as low as 10^2^ CFU mL^−1^ spectrophotometrically and 10^4^ CFU mL^−1^ by the naked eye, which is compatible with the current clinical setup. Based on these observations, we anticipate that this colorimetric bacteria detection assay developed using the specific affinity and catalytic activity of CS-MNPs may provide a new way to detect broad-spectrum bacteria on site.

## Supplementary Materials

The following figures are available online at https://www.mdpi.com/2079-4991/10/1/92/s1, Figure S1: Zeta potential analyses of CS-MNPs prepared with different concentrations of chitosan, Figure S2: Comparison of the peroxidase-like activity between MNPs and CS-MNPs, Figure S3: Zeta potential analyses of free CS only, bacteria only, and bacteria with free CS. *E. coli* (EC) and *S. aureus* (SA) were used in the experiments.
