# Supplementary material for: A Convenient Colorimetric Bacteria Detection Method Utilizing Chitosan-Coated Magnetic Nanoparticles
Source: Nanomaterials (Basel). 2020 Jan 2;10(1):92. doi: 10.3390/nano10010092 (PMC7023617; doi:10.3390/nano10010092)
Supplement: Supplementary file 1 [file nanomaterials-10-00092-s001.pdf]

nanomaterials

Supplementary Materials

A Convenient Colorimetric Bacteria Detection Method Utilizing Chitosan-Coated Magnetic Nanoparticles

Thao Nguyen Le, Tai Duc Tran and Moon Il Kim*

Department of BioNano Technology, Gachon University, 1342 Seongnamdae-ro, Sujeong-gu, Seongnam, Gyeonggi 13120, Republic of Korea; thaonguyen65949@gmail.com (T.N.L); tdtai.151294@gmail.com (T.D.T)

* Correspondence moonil@gachon.ac.kr; Tel.: +82-31-750-8563

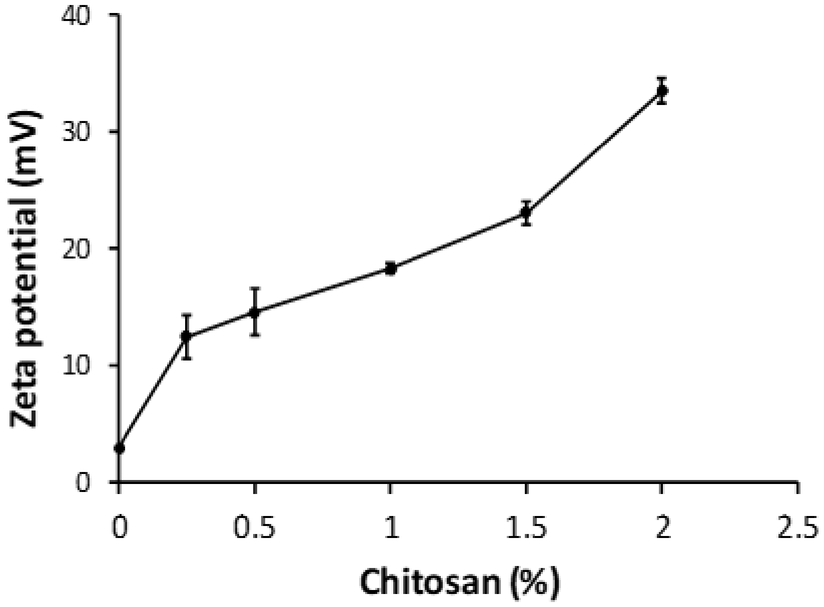

Figure S1. Zeta potential analyses of CS-MNPs prepared with different chitosan concentrations.

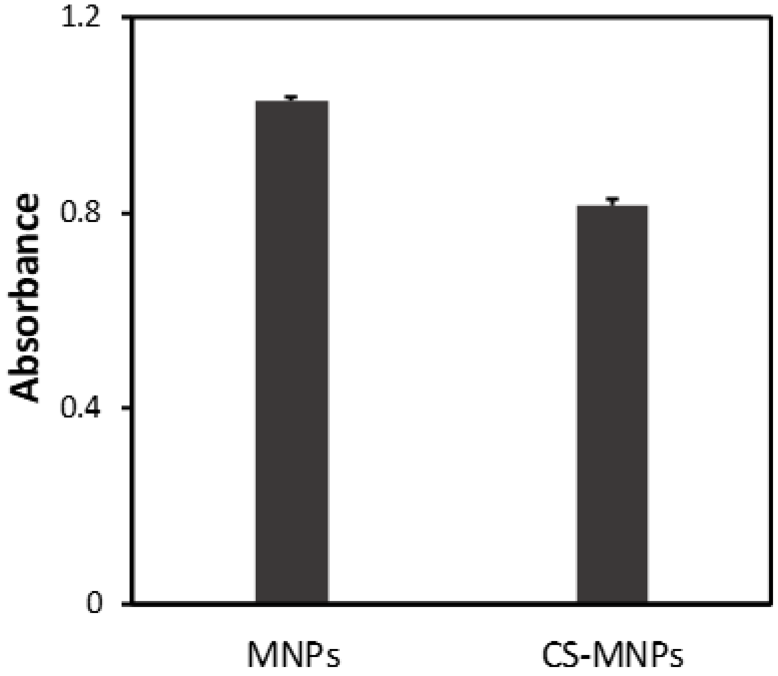

Figure S2. Comparison of the peroxidase-like activity between MNPs and CS-MNPs.

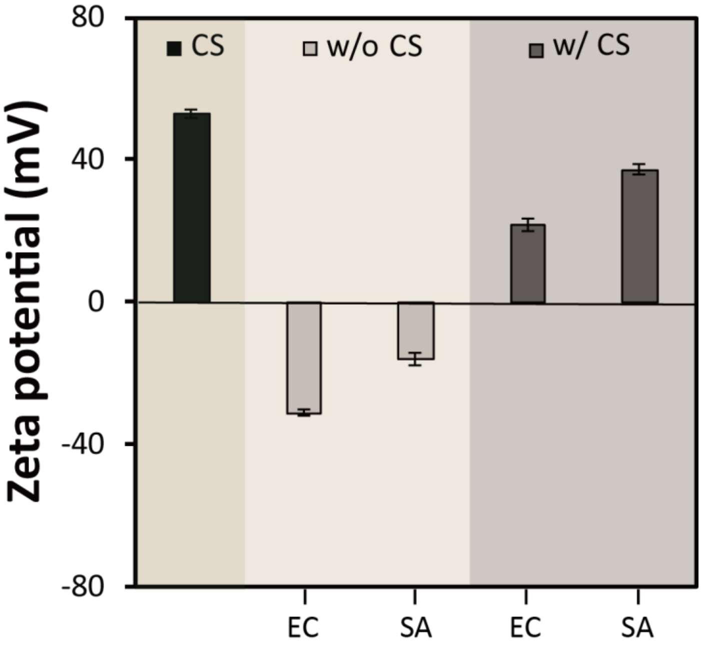

Figure S3. Zeta potential analyses of free CS only, bacteria only, and bacteria with free CS. *E. coli* (EC) and *S. aureus* (SA) were used in the experiments.

© 2020 by the authors. Submitted for possible open access publication under the terms and conditions of the Creative Commons Attribution (CC BY) license (http://creativecommons.org/licenses/by/4.0/).
